# Identifying an lncRNA-Related ceRNA Network to Reveal Novel Targets for a Cutaneous Squamous Cell Carcinoma

**DOI:** 10.3390/biology10050432

**Published:** 2021-05-13

**Authors:** Yaqin Xu, Yingying Dong, Yunhua Deng, Qianrong Qi, Mi Wu, Hongmei Liang, Qiuyun She, Qing Guo

**Affiliations:** 1 Department of Dermatology, Tongji Medical College, Huazhong University of Science and Technology, Wuhan 430030, China; xuyaqin@hust.edu.cn (Y.X.); yingyingdong@hust.edu.cn (Y.D.); qiuyunshe@hust.edu.cn (Q.S.); 2 Department of Obstetrics & Gynecology, University of California, Irvine, CA 92697, USA; 3 Department of Immunology, School of Basic Medicine, Tongji Medical College, Huazhong University of Science and Technology, Wuhan 430030, China; wumi@hust.edu.cn (M.W.); lianghongmei@hust.edu.cn (H.L.); qingguo@hust.edu.cn (Q.G.)

**Keywords:** competing endogenous RNAs network, long non-coding RNAs, microRNAs, cutaneous squamous cell carcinoma

## Abstract

**Simple Summary:**

The exact functions and molecular mechanism of lncRNAs, acting as competitive endogenous RNAs in a cutaneous squamous cell carcinoma, remain unexplored. The present study was conducted to identify the differentially expressed lncRNAs and mRNAs and establish the lncRNA-related competing endogenous RNA networks associated with a cutaneous squamous cell carcinoma. A competing endogenous RNA network consisting of 137 miRNA-lncRNA and 221 miRNA-mRNA pairs was constructed. As for the functional analysis of the mRNAs in the network, a FoxO signaling pathway, an autophagy and cellular senescence were the top enrichment terms based on the Kyoto Encyclopedia of Genes and Genomes analysis. We identified five core mRNAs and built a core mRNA-associated competing endogenous RNA network. Finally, one lncRNA HLA-F-AS1 and three mRNAs named AGO4, E2F1 and CCND1 in the core mRNA-associated competing endogenous RNA network were validated with the same expression patterns. The core mRNAs and their associated lncRNAs may provide potential therapeutic targets for cutaneous squamous cell carcinomas.

**Abstract:**

A cutaneous squamous cell carcinoma (cSCC) derived from keratinocytes is the second most common cause of non-melanoma skin cancer. The accumulation of the mutational burden of genes and cellular DNA damage caused by the risk factors (e.g., exposure to ultraviolet radiation) contribute to the aberrant proliferation of keratinocytes and the formation of a cSCC. A cSCC encompasses a spectrum of diseases that range from recursor actinic keratosis (AK) and squamous cell carcinoma (SCC) in situ (SCCIS) to invasive cSCCs and further metastatic SCCs. Emerging evidence has revealed that lncRNAs are involved in the biological process of a cSCC. According to the ceRNA regulatory theory, lncRNAs act as natural miRNA sponges and interact with miRNA response elements, thereby regulating the mRNA expression of their down-stream targets. This study was designed to search for the potential lncRNAs that may become potential therapeutic targets or biomarkers of a cSCC. Considering the spirit of the study to be adequately justified, we collected microarray-based datasets of 19 cSCC tissues and 12 normal skin samples from the GEO database (GSE42677 and GSE45164). After screening the differentially expressed genes via a limma package, we identified 24 differentially expressed lncRNAs (DElncRNAs) and 3221 differentially expressed mRNAs (DEmRNAs). The miRcode, miRTarBase, miRDB and TargetScan databases were used to predict miRNAs that could interact with DElncRNAs and DEmRNAs. A total of 137 miRNA-lncRNA and 221 miRNA-mRNA pairs were retained in the ceRNA network, consisting of 31 miRNAs, 11 DElncRNAs and 155 DEmRNAs. For the functional analysis, the top enriched biological process was enhancer sequence-specific DNA binding in Gene Ontology (GO) terms. The FoxO signaling pathway, autophagy and cellular senescence were the top enrichment terms based on a Kyoto Encyclopedia of Genes and Genomes (KEGG) analysis. The combination of a STRING tool and Cytoscape software (plug-in MCODE) identified five core mRNAs and built a core mRNA-associated ceRNA network. The expression for five identified core mRNAs and their related nine lncRNAs was validated using the external dataset GSE7553. Finally, one lncRNA HLA-F-AS1 and three mRNAs named AGO4, E2F1 and CCND1 were validated with the same expression patterns. We speculate that lncRNA HLA-F-AS1 may sponge miR-17-5p or miR-20b-5p to regulate the expression of CCND1 and E2F1 in the cSCC. The present study may provide potential diagnostic and therapeutic targets for cSCC patients.

## 1. Introduction

Cutaneous squamous cell carcinoma (cSCC), which originates from epidermal keratinocytes, ranks as the second most occurring skin cancer [1]. The occurrence of cSCC is due to many risk factors. Beside the most important risk factor, cumulative ultraviolet exposure, other risk factors include old age, immunosuppression, smoking and genetic factors [2]. Moreover, a cSCC commonly has high mutational burden (e.g., TP53, CDKN2A, Ras and NOTCH1). The accumulation of these mutations and cellular DNA damage caused by the risk factors contribute to the uncontrolled proliferation of keratinocytes and the formation of a cSCC and disease progression [3]. Consequently, a cSCC is a spectrum of diseases that commonly arises from precursor actinic keratosis (AK) and a squamous cell carcinoma (SCC) in situ (SCCIS) to an invasive cSCC to a metastatic SCC with a distant spread [4]. Based on the tumor stage, the optimal treatment approach for a cSCC is selected. The cSCC at the early stage is generally identified and can be cured via surgical resection and/or radiotherapy. For cases at the advanced stage (invasive and metastatic cSCCs), a single or a combined therapy, including chemotherapy, radiation therapy and epidermal growth factor receptor (EGFR) inhibitors, have been implemented with limited and unsatisfactory efficacy with a long-term survival rate of less than 20% [4]. Thus, it is urgent to identify new biomarkers and targets that are more effective for the early diagnosis and treatment of a cSCC.

Non-coding RNAs (ncRNAs) incapable of protein coding can be classified according to their length including long non-coding RNAs (lncRNAs) (>200 nucleotides) and small ncRNAs (<200 nucleotides) such as microRNAs (miRNAs), small nucleolar RNAs and transfer RNAs [5]. The dysregulation of the expression of miRNAs has also been elucidated to be implicated in the tumorigenesis of a cSCC. The upregulated miR-142-5p induced cancer stem cell-like properties in a cSCC via the direct regulation of PTEN [6]. MiR-27a directly bound to EGFR, leading to the inactivation of the NF-κB pathway and the inhibition of cSCC cell growth [7].

LncRNAs are a wide and diverse class of single-stranded ncRNAs longer than 200 nucleotides lacking a protein-coding capacity [8]. LncRNA dysregulation has also been linked to the process of cell differentiation and growth and the pathogenesis of many types of cancers [9,10] including cSCCs [11,12]. LINC00520 targeted EFGR to inactivate the PI3K/Akt pathway and restrain the cSCC progress [11]. The lncRNA PICSAR dysregulated the collagen and fibronectin expression, resulting in the dysregulation of the adhesion and migration of cSCC cells [12]. Emerging evidence demonstrates that activities of lncRNAs and miRNAs are related to each other in different complex mechanisms [13]. One of these mechanisms is the role of lncRNAs as competing endogenous RNAs (ceRNAs). Specifically, lncRNAs can compete with mRNA to bind miRNA binding sites, thereby negatively regulating miRNAs and their target genes. In other words, lncRNAs act as a sponge for miRNAs abolishing their inhibitory action to target mRNAs [14]. The lncRNA-miRNA-mRNA regulatory network has been verified to be associated with the progression of several types of squamous cell carcinomas (SCCs) [15,16,17]. LncRNA nuclear paraspeckle assembly transcript 1 (NEAT1) sponged to miR-129, leading to the depression of CTBP2 and regulated the esophageal squamous cell carcinoma (ESCC) cell viability and invasion [15]. LINC00511 induced the expression of LAMC2 via sponging to miR-765 and modulated a tongue squamous cell carcinoma progression (TSCC) [16]. Zhao et al. indicated that lncRNA HCP5 functioned as a molecular sponge to absorb miR-140-5p in an oral squamous cell carcinoma (OSCC) and upregulated the SOX4 gene expression [17]. As for cSCCs, lncRNA HOTAIR promoted the progression of a cSCC, exerting a function as a ceRNA through the miR-326/Prenylated Rab acceptor 1 domain family member 2 (PRAF2) axis [18].

In this paper, we compared total RNA expression data based on an array between cSCC and normal skin (NS) samples from the National Center of Biotechnology Information (NCBI) Gene Expression Omnibus (GEO) database. Based on the differentially expressed genes (DEGs), we performed a series of down-stream analyses including the construction of an lncRNA-related ceRNA network, a functional enrichment analysis and a protein-protein interaction analysis. We theorize that lncRNA HLA-F-AS1 may act as the sponge of miR-17-5p and miR-20b-5p to regulate the expression of CCND1 and E2F1 in the cSCC. The expression of HLA-F-AS1 in the cSCC samples and its role mechanism in the biological process of a cSCC as a ceRNA required further research. They may be candidate targets with a promising diagnostic or therapeutic value with a cSCC.

The remainder of this paper is organized as follows. Section 2 introduces the dataset collection and presents our designed data processing workflow and describes our down-stream analyses and validation methods. The experiment results are described in Section 3. The Discussion based on the experiment results is analyzed in Section 4. Conclusions are drawn in Section 5.

## 2. Materials and Methods

### 2.1. Dataset Collection from the GEO Database

The expression profiles of lncRNAs and mRNAs between a cSCC and normal skin (NS) from healthy samples were downloaded from the GEO database (www.ncbi.nlm.nih.gov/geo, accessed on 24 August 2020). The criterion for selecting the cSCC samples was that the clinical and histological diagnosis of biopsies obtained during surgery was a primary cSCC (SCCIS or invasive cSCC). The datasets were recruited with the standard based on the same microarray platform and containing more than three samples in each group. Finally, GSE42677 and GSE45164 dataset bases on the GPL571 platform were obtained including 20 cSCC and 13 NS samples.

### 2.2. Data Preprocessing and Screening for DElncRNAs and DEmRNAs

**Figure 1:**
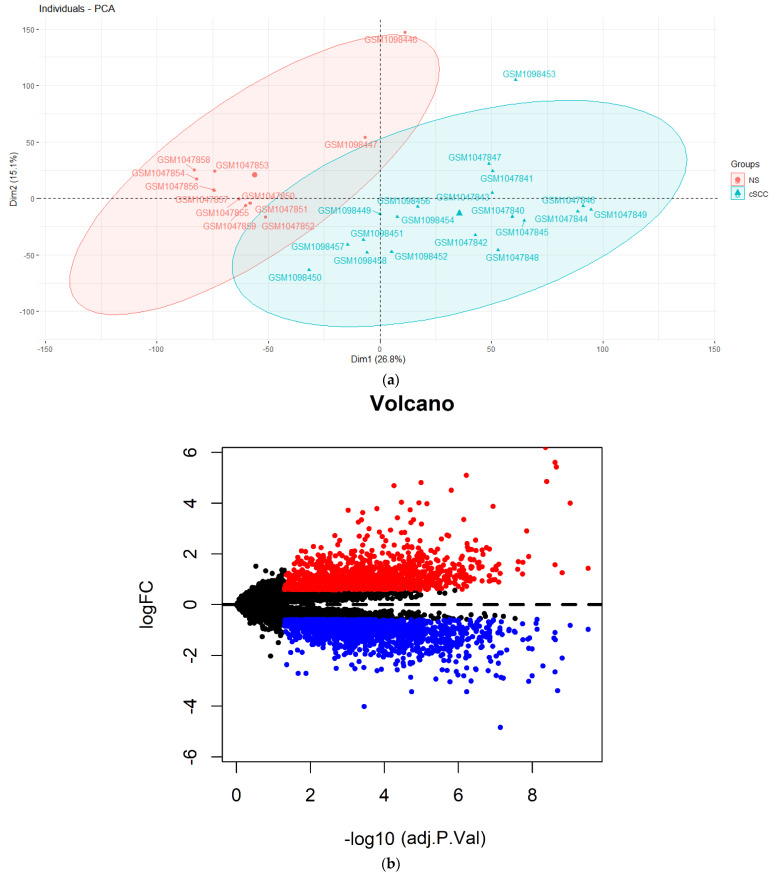

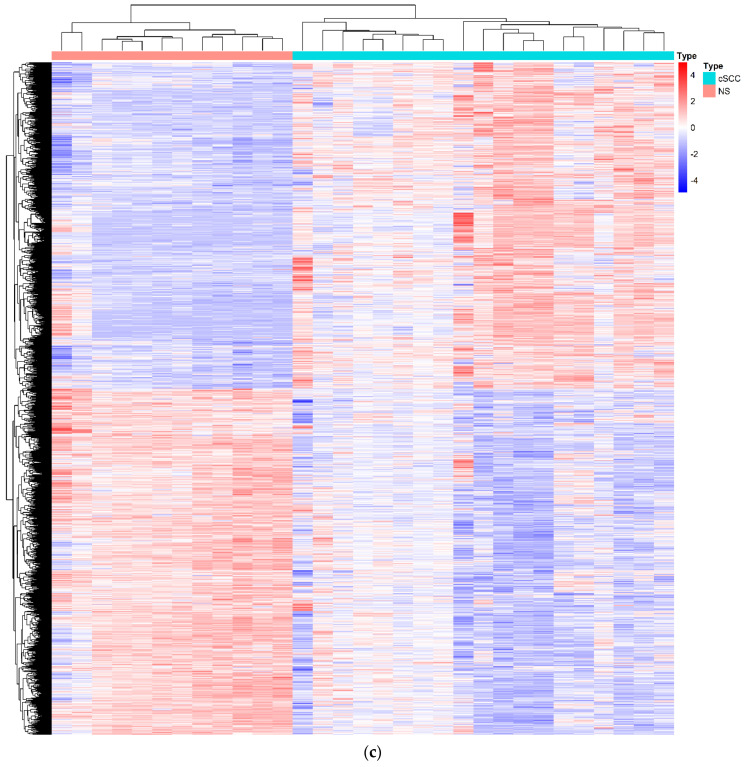
Differentially expressed analysis between cSCC and NS tissues from GSE42677 and GSE45164 datasets. (**a**) The principal component analysis (PCA) of the selected 19 cSCC and 12 NS tissues; (**b**) volcano plots showing DEGs. The red dots represent an upregulated expression and the blue dots represent downregulated DEGs; (**c**) cluster analysis (heatmap) between 19 cSCC and 12 NS tissues. The red stripes display upregulation while the blue stripes display the downregulation of genes. DEGs: differential expression genes.

Based on the annotation information from Affymetrix Human Genome U133A 2.0 Array platform, the probe sequences were quantified to the expression of lncRNAs and mRNAs after mapping to the human GRCh38 reference genome (http://useast.ensembl.org/info/data/ftp/index.html, accessed on 29 August 2020). We integrated all samples from the two datasets (GSE42677 and GSE45164) and applied ComBat [19] to remove the batch-effects of expression values (Figure S1a,b). A principal component analysis (PCA) [20] was applied to assess the independence of the available genes between 20 cSCC and 13 NS samples. According to the PCA result (Figure S1), we removed one cSCC and one control sample affecting the classification. Data of 19 cSCC patients and 12 NS were selected for a subsequent analysis (Figure 1a).

A Limma package [21] (version 3.44.3; https://bioconductor.org/packages/release/bioc/html/limma.html, accessed on 29 August 2020) in R was applied to identify the differentially expressed lncRNAs (DElncRNAs) and differentially expressed mRNAs (DEmRNAs) before a quantile normalization and base-2 logarithmic (log_2_) transformation. The criteria were selected as a false discovery rate (FDR) < 0.05 and a fold change (FC) > 1.5. Volcano plots were performed for the DEGs using the ggplot2 package [22] (version 3.3.2; https://cran.r-project.org/web/packages/ggplot2/index.html, accessed on 29 August 2020) in the R software. Pheatmap package (version: 1.0.12; https://cran.r-project.org/web/packages/pheatmap/, accessed 29 August 2020) in R was used to generate the hierarchical cluster heatmap representing the expression intensity and direction (Figure 1b).

### 2.3. Construction of the cSCC-Associated lncRNA-miRNA-mRNA Network

Due to the absence of miRNA information, the miRcode database [23] (http://www.mircode.org/, accessed on 1 September 2020) was used to predict the targeted miRNAs of DElncRNAs. According to the miRTarBase [24] (http://mirtarbase.mbc.nctu.edu.tw/, accessed on 1 September 2020), miRDB [25] (http://www.mirdb.org/, accessed on 1 September 2020) and TargetScan [26] (http://www.targetscan.org/), the DEmRNA-miRNA interaction pairs were obtained. Only the overlapping of the interaction pairs supported by all databases was retained to create the miRNA-mRNA pairs. Subsequently, the correlation between DElncRNAs and DEmRNAs was analyzed by Pearson’s correlation coefficient (PCC) with the threshold of the PCC value r > 0.4 and *p* < 0.05 indicating a strong correlation. Finally, the remaining miRNA-DElncRNA and miRNA-DEmRNA and their shared miRNAs were incorporated into the ceRNA network. Cytoscape [27] (version 3.6.1; http://cytoscape.org, accessed on 6 September 2020) was utilized to visualize the network.

### 2.4. Functional Enrichment Analysis

The Gene Ontology (GO) functional enrichment analysis in the category Biological Processes (BP) and the KEGG pathway enrichment analysis of DEmRNAs in the ceRNA network were determined by the clusterProfiler package in R [28] (version 3.14.0; https://bioconductor.org/packages/release/bioc/html/clusterProfiler.html, accessed on 12 September 2020) and R. The threshold was selected to be *p* < 0.05.

### 2.5. Establishment of the Protein-Protein Interaction (PPI) Network and the Core ceRNA Subnetwork

The Search Tool for the Retrieval of Interacting Genes (STRING) [29] (http://string-db.org/, accessed on 12 September 2020) was utilized to analyze PPI information with the threshold of a combined score >0.4. The plug-in Molecular Complex Detection (MCODE; version:1.4.2; http://apps.cytoscape.org/apps/mcode, accessed on 12 September 2020) [30] in Cytoscape was utilized to identify functionally-related hub modules with a degree cut-off of more than 5, a node score cut-off of 0.2, a k-score of 2 and maximum depth of 100. The key genes and related lncRNAs and miRNAs were then mapped into the core ceRNA subnetwork.

### 2.6. Expression Validation in an External Dataset

To validate the expression of the DElncRNAs and DEmRNAs in the subnetwork, the GSE7553 based on the GPL570 platform with 11 SCC and 4 NS samples was obtained from GEO. The inclusion criteria of the cSCC samples, the data processing of the identifying DElncRNAs and DEmRNAs of GSE7553 datasets and the cut-off criteria were consistent with those of the GSE42677 and GSE45164 datasets. If the relative expression level of genes in the core ceRNA network was significantly different and met the same expression patterns with the GSE42677 and GSE45164 datasets, we considered those genes validated. The comparison of the log_2_ transformed expression level of the DElncRNAs and DEmRNAs was performed by GraphPad Prism V8.3.0 software (GraphPad Software, Inc., San Diego, CA, USA) with an unpaired t-test and *p*  <  0.05 indicating a significant difference.

## 3. Results

### 3.1. A Total Of 24 DElncRNAs and 3221 DEmRNAs Were Identified between cSCC and NS

Based on the PCA results of all samples from the GSE42677 and GSE45164 datasets, one cSCC sample and one NS sample affecting the classification were excluded for the down-stream analyses (Figure S1c). The heatmap of the DEGs showed that the gene expression levels between the remaining 19 cSCC and 12 NS samples were completely distinguishable (Figure 1c). Compared with the NS samples, 24 DElncRNAs (18 over-expressed and 6 down-expressed) and 3221 DEmRNAs (1568 over-expressed and 1653 down-expressed) were identified with the threshold of FDR < 0.05 and |FC| > 1.5.

### 3.2. The cSCC-Associated ceRNA Network Establishment

**Figure 2:**
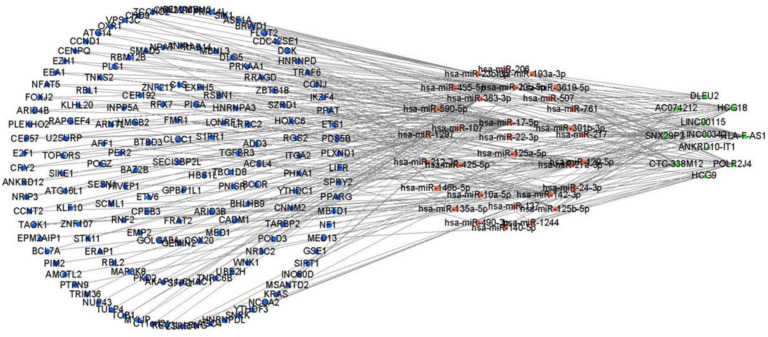
The construction of the ceRNA network including 11 DElncRNAs, 31 predicted miRNAs and 155 DEmRNAs. The circle in green, the rectangle in red and the triangle in blue represent DEmRNAs, miRNAs and DElncRNAs, respectively. DElncRNAs: differentially expressed lncRNAs; DEmRNAs: differentially expressed mRNAs; miRNAs: microRNAs.

Before predicting the miRNAs interacting with DElncRNAs or DEmRNAs, the co-expression analysis by the PCC was applied to calculate the correlation between DElncRNA and DEmRNA. Based on the result of the PCC with a cut-off value r > 0.4 and *p* < 0.05, 155 DEmRNAs and 23 DElncRNAs were retained for a further analysis (Table S1). The remaining DEmRNAs and DElncRNAs that shared the common miRNAs were incorporated into the construction of the ceRNA network (Figure 2). Finally, a cSCC-associated ceRNA network containing 137 miRNA-lncRNA (Table S2) and 221 miRNA-mRNA (Table S3) pairs was created composed of 31 miRNAs, 11 DElncRNAs (8 down-expressed and 3 over-expressed, Table S4) and 155 DEmRNAs (130 down-expressed and 25 over-expressed) (Tables S4 and S5).

### 3.3. DEmRNAs of the ceRNA Network Were Associated with Enhancer-Binding, Protein Kinase Regulator Activity and Autophagy

**Figure 3:**
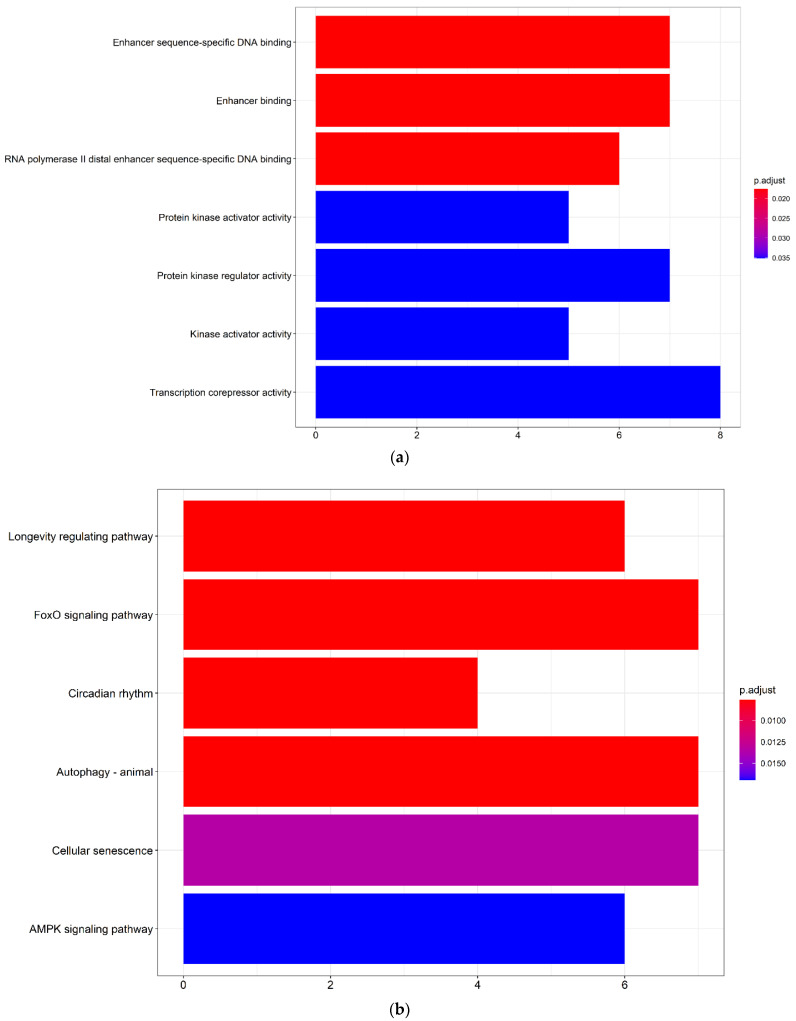
Bar chart of the GO and KEGG analysis of DEmRNAs in the ceRNA network. (**a**) Bar chart of the significant GO terms with DEmRNAs identified by the clusterProfiler package in R. The color of the bar represents the −log_10_ transformed *p* values of the GO terms and the abscissa corresponds with the number of genes in the GO terms; (**b**) bar chart of significant KEGG enriched pathways with DEmRNAs identified by the clusterProfiler package in R. The color of the bar represents the −log10 transformed *p* values of the KEGG enriched pathways and the abscissa corresponds with the number of genes in the KEGG enriched pathways. GO: Gene Ontology; KEGG: Kyoto Encyclopedia of Genes and Genomes; DElncRNAs: differentially expressed lncRNAs; DEmRNAs: differentially expressed mRNAs.

To uncover the biological mechanisms associated with the development of a cSCC, a GO functional and a KEGG pathway enrichment analysis of the DEmRNAs in the ceRNA network were performed. In the GO enrichment analysis (Figure 3a, Table S6), the DEmRNAs were involved in the biological processes of enhancer sequence-specific DNA binding, enhancer-binding, protein kinase regulator activity and transcription corepressor activity. The significant KEGG pathway (Figure 3b, Table S7) enrichment included the forkhead box O (FoxO) signaling pathway, autophagy and cellular senescence.

### 3.4. PPI Network and the Core ceRNA Subnetwork Construction

**Figure 4:**
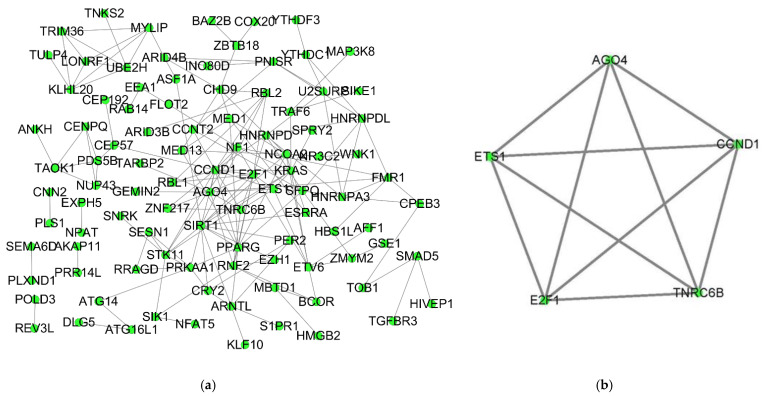
PPI network of DEmRNAs and Cluster 1 using the MCODE plug-in from the whole PPI network. (**a**) PPI network of DEmRNAs in the ceRNA network obtained from the STRING database. (**b**) The first cluster is of PPI followed by the MCODE plug-in of Cytoscape. PPI: Protein-protein interaction; DEmRNAs: differentially expressed mRNAs; MCODE: molecular complex.

**Figure 5:**
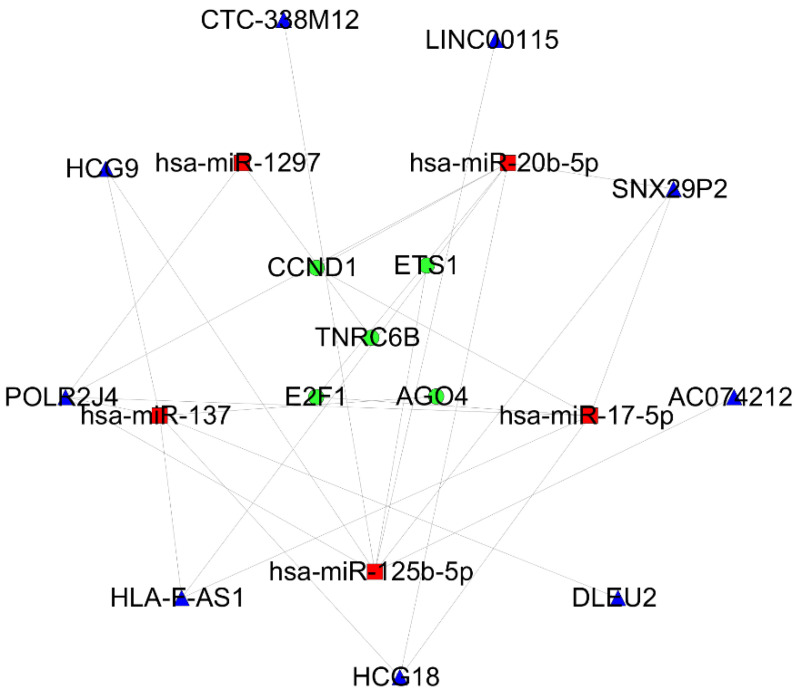
The hub-genes associated with the ceRNA network in a cSCC. MRNAs, miRNAs and lncRNAs are indicated by the circle in green, rectangle in red and triangle in blue, respectively.

The combination of the STRING tool and Cytoscape software constructed the PPI network. A total of 99 nodes and 170 interactions were screened from the PPI network. After a MCODE analysis, five hub-genes were incorporated into the PPI network (Figure 4a,b). Based on the ceRNA network, the hub-genes associated with the core ceRNA subnetwork (Tables S8 and S9) were established including 20 miRNA-lncRNA and 7 miRNA-mRNA edges and 19 nodes (9 lncRNAs (HCG9, POLR2J4, HLA-F-AS1, HCG18, DLEU2, AC074212, SNX29P2, LINC00115 and CTC-338M12), 5 miRNAs (hsa-mir-1297, hsa-mir-137, hsa-mir-17-5p, hsa-mir-20b-5p and hsa-mir-25b-5p) and 5 mRNAs (AGO4, E2F1, CCND1, TNRC6B and ETS1)) (Figure 5).

### 3.5. Verification of the DEGs in the Core ceRNA Subnetwork

**Figure 6:**
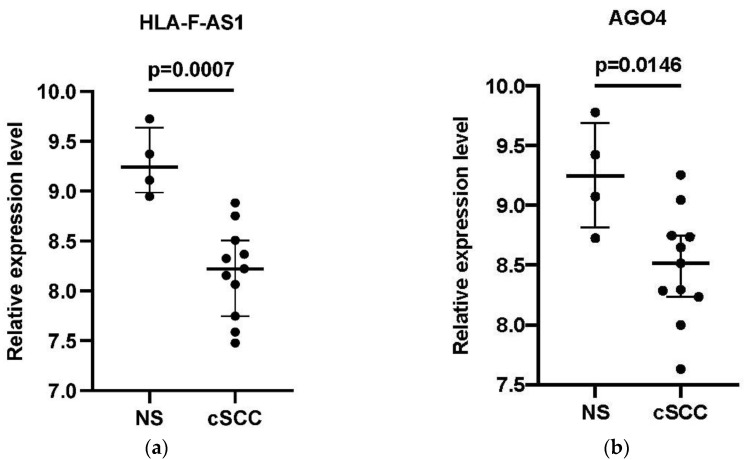

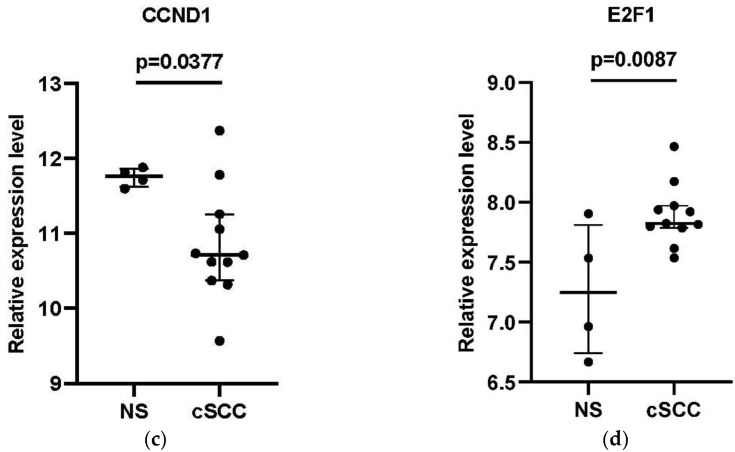
External validation of the four core genes in the GSE7553 datasets. (**a**) The relative expression level of HLA-F-AS1 between cSCC and NS samples; (**b**) the relative expression level of AGO4 between cSCC and NS samples; (**c**) the relative expression level of CCND1 between cSCC and NS samples; (**d**) the relative expression level of E2F1 between cSCC and NS samples. The log_2_ transformed expression level corresponded with the relative expression level. Data are presented as a median with interquartile ranges. An unpaired t-test was used for the comparison of the relative expression level between the two groups.

The expression of 9 DElncRNAs and 5 DEmRNAs in the hub-genes associated with the core ceRNA subnetwork were compared between the cSCC and the NS group using the GSE7553 dataset. Finally, one lncRNA HLA-F antisense RNA 1 (HLA-F-AS1) and 3 DEmRNAs (Argonaute-4 (AGO4), Cyclin D1 (CCND1) and E2F transcription factor 1 (E2F1)) were considered to be validated of which the expression patterns were consistent with the results of GSE42677 and GSE45164 (Figure 6).

## 4. Discussion

A cutaneous squamous cell carcinoma (cSCC) derived from the malignant proliferation of keratinocytes is the second most frequent cause of non-melanoma skin cancer [31]. However, a cSCC usually displays a primary cSCC with benign clinical behavior and can be successfully treated with surgery. It may progress to be locally invasive and metastatic [32]. The etiology and pathogenesis of a cSCC have not been well elucidated. Recently, accumulating studies have emphasized the regulatory role of lncRNAs as ceRNAs in the occurrence and progression of many types of cancers including cSCCs [15,16,17,18]. We then constructed an lncRNA-associated ceRNA network based on the transcriptome expression profiles from the GEO database to identify new targets with a potential diagnostic or curative value for the cSCC.

In total, 24 DElncRNAs and 3221 DEmRNAs were identified between the cSCC tumors compared with NS samples. The DElncRNAs and DEmRNAs with a strong relationship were incorporated with their common targeted miRNAs to construct the ceRNA network. The DEmRNAs in the ceRNA network were then subjected to the GO enrichment and KEGG pathway enrichment analyses. The pathway analysis showed that the AMPK signaling pathway [33] and autophagy [34] participate in the pathogenesis of a cSCC.

We established a hub-gene-associated core ceRNA subnetwork including nine lncRNAs, five predicted miRNAs (hsa-mir-1297, hsa-mir-137, hsa-mir-17-5p, hsa-mir-20b-5p and hsa-mir-25b-5p) and five mRNAs (AGO4, E2F1, CCND1, TNRC6B and ETS1). Three DEmRNAs (E2F1, CCND1 and AGO4) were validated in the independent dataset GSE7553. E2F1 and CCND1 are both cell cycle regulatory proteins. The upregulated expression of E2F1 and its family number E2F7 in cSCCs may contribute to cSCC formation [35]. E2F1 was shown to inhibit the differentiation of keratinocytes at the terminal phase and inactivate MMP [36,37]. The aberrant activation of E2F1 also participated in the cellular proliferation, differentiation and apoptosis of colon cancer and prostate cancer and is often related to a poor prognosis [38,39]. CCND1 has been discovered to be upregulated in SCCs such as laryngeal squamous cell carcinomas (LSCCs), head and neck squamous cell carcinomas (HNSCCs) and oral squamous cell carcinomas (OSCCs) [40,41,42]. As for the AGO4, it may exert a function as an effector protein with DNA methylation via RNA-dependent DNA methylation (RdDM) [43]. A DNA methyltransferase 3A (DNMT3A)/AGO4 complex abolished miR-181-5p inhibition of gene expression via the cytosine methylation of miR-181-5p and resulted in an aggressive outcome of a glioblastoma multiforme (GBM) [44].

Among the five predicted miRNAs, miR-17-5p had been validated to be highly and significantly upregulated in a cSCC by a real-time polymerase chain reaction (RT-PCR) between cSCC tissues and NS samples [45]. MiR-17-5p and miR-20b-5p are both members of the miR-17-92 family. Moreover, the interaction of miR-17 with CCND1 and E2F1 has been studied. In a study of a lung adenocarcinoma, miR-20b-5p was found to directly and independently inhibit the activity of reporter genes and decrease the CCND1 protein expression [46]. MiR-20b-5p could sponge with the 3′ untranslated region (3′ UTR) of CCND1, repressing the CCND1 expression in a lung cancer cell [46]. MiR-20b-5p also targeted with E2F1, inducing a myoblast differentiation and repressing its proliferation [47].

Only one lncRNA HLA-F-AS1 in the core ceRNA subnetwork was validated in the external datasets. Several studies had revealed the dysregulation of HLA-F-AS1 in cancers [48,49,50] while the transcript expression of HLA-F-AS1 was verified to be downregulated in lung adenocarcinoma (LAD) samples [49]. It was over-expressed in colorectal cancer (CRC) tissues and CRC cell lines. Huang et al. found that HLA-F-AS1 may participate in the progression of CRC via the miR-330-3p/profilin 1 (PFN1) axis in CRC cells [48]. The elevated HLA-F-AS1 expression induced by STAT3 promoted cell growth and stemness characteristics via sponging with miR-541-3p to upregulate the expression of a TraB domain (TRABD) containing triple-negative breast cancer cells [50].

In summary, according to the predicted core ceRNA subnetwork, lncRNA HLA-F-AS1 may sponge miR-17-5p or miR-20b-5p to regulate the expression of CCND1 and E2F1 in the cSCC. The expression of HLA-F-AS1 in the cSCC samples and its role mechanism in the biological process of a cSCC as a ceRNA required further research. To highlight the main findings of the validated lncRNAs and miRNAs in the core ceRNA subnetwork, we followed the model presented by Garofoli et al. [51] and made a table where a systematic review of the roles and mechanisms of HLA-F-AS1, miR-17-5p and miR-20b-5p in the pathogenesis of cancers was carried out (Table 1).

## 5. Conclusions

In this study, the cSCC-related lncRNA-miRNA-mRNA ceRNA network was established and one lncRNA (HLA-F-AS1) and three mRNAs (AGO4, CCND1 and E2F1) in the core ceRNA network were validated. The HLA-F-AS1-miR-17-5p/miR-20b-5p-CCND1/E2F1 axis may take part in the development of a cSCC. However, there are several limitations in our research that should be considered. First, miRNA expression datasets are needed to verify the network. Second, a relatively large sample size should be collected to assure the results are more reliable in future studies. Moreover, the relation between the ceRNA network and clinical features could not be established due to the absence of related clinical information. Our research provides a new therapeutic idea and regulatory roles of ceRNAs in the pathogenesis of a cSCC. They may be candidate targets with a promising diagnostic or therapeutic value with a cSCC. Further functional investigations mediated by the ceRNA would have to be verified.

## Figures and Tables

**Table 1:** Summary of the roles and mechanisms of discussed lncRNAs and miRNAs in the pathogenesis of cancers.

LncRNA/miRNA	Gene Targets	Mechanisms in Cancers
HLA-F-AS1	MiR-541-3p	Promotion of cell proliferation and stemness in vitro and tumor growth in vivo by the miR-541-3p/TRABD axis in triple-negative breast cancer [50]. Promotion of cell proliferation, migration and invasion and repression of apoptosis in vitro via the miR-330-3p/ PFN1 axis in colorectal cancer [48].
	MiR-330-3p	
MiR-17-5p	3′ UTR of CCND1	Repression of the CCND1 protein expression via sponging with the CCND1 3′-UTR region in a lung cancer cell [46]. Promotion of cell growth, proliferation, migration and colony formation in vitro in a non-metastatic hepatocellular carcinoma by regulating E2F1 expression [52].
	E2F1	
MiR-20b-5p	3′ UTR of CCND1	Promotion of myoblast differentiation and repression of myoblast proliferation by regulating E2F1 expression [47]. Promotion of cell proliferation, G1/S transition and DNA synthesis by downregulating CCND1 and E2F1 expression in multipotent stromal cells [53]. Promotion of cell migration and proliferation and repression of apoptosis of breast cancer stem cells and promoting tumor growth in vivo by bidirectionally regulating the protein levels of CCND1 and E2F1 in breast cancer [54]. Inhibition of the cell cycle, migration and invasion in vitro and the tumorigenesis in vivo by negatively regulating CCND1 in colon cancer [55].
	3′ UTR of E2F1	

HLA-F-AS1: HLA-F antisense RNA 1; CCND1: Cyclin D1; E2F1: E2F transcription factor 1; TRABD: TraB domain containing; PFN1: profilin 1; 3′ UTR: 3′ untranslated regions.

## Data Availability

Data were obtained from the GEO database and are available at: www.ncbi.nlm.nih.gov/geo (accessed on 24 August 2020) with the permission of the GEO database.

## Supplementary Materials

The following are available online at https://www.mdpi.com/article/10.3390/biology10050432/s1, Figure S1: Data preprocessing. Table S1: The lncRNA-mRNA pairs with significant correlations, Table S2: DElncRNA-miRNA pairs of the ceRNA network, Table S3: DEmRNA-miRNA pairs of the ceRNA network, Table S4: Over-expressed and down-expressed lncRNAs of the ceRNA network, Table S5: Over-expressed and down-expressed mRNAs of the ceRNA network, Table S6: GO enrichment terms of DEmRNAs in the ceRNA network, Table S7: KEGG pathway enrichment of DEmRNAs in the ceRNA network, Table S8: Details of DElncRNA-miRNA pairs of the core ceRNA network, Table S9: DEmRNA-miRNA pairs of the core ceRNA network.

## Abbreviations

SCC	Squamous Cell Carcinoma
cSCC	Cutaneous Squamous Cell Carcinoma
SCCIS	Squamous Cell Carcinoma (SCC) In Situ
NcRNAs	Non-Coding RNAs
LncRNAs	Long Non-Coding RNAs
MiRNAs	MicroRNAs
CeRNAs	Competing Endogenous RNAs
NS	Normal Skin
DEGs	Differentially Expressed Genes
DElncRNAs	Differentially Expressed LncRNAs
DEmRNAs	Differentially Expressed MRNAs
GEO	Gene Expression Omnibus
GO	Gene Ontology
BP	Biological Processes
KEGG	Kyoto Encyclopedia of Genes and Genomes
STRING	Search Tool for the Retrieval of Interacting Genes
MCODE	Molecular Complex Detection
FDR	False Discovery Rate
FC	Fold Change
PCA	Principal Component Analysis
PCC	Pearson’s Correlation Coefficient
CDC20	Cell Division Cycle 20
EGFR	Epidermal Growth Factor Receptor
PRAF2	Prenylated Rab Acceptor 1 Domain Family Member 2
FoxO	Forkhead box O
HLA-F-AS1	HLA-F Antisense RNA 1
CCND1	Cyclin D1
E2F1	E2F Transcription Factor 1
AGO4	Argonaute-4
LAD	Lung Adenocarcinoma
CRC	Colorectal Cancer
PFN1	Profilin 1
ESCC	Esophageal Squamous Cell Carcinoma
TSCC	Tongue Squamous Cell Carcinoma Progression
LSCC	Laryngeal Squamous Cell Carcinoma
HNSCC	Head and Neck Squamous Cell Carcinoma
OSCC	Oral Squamous Cell Carcinoma
RdDM	RNA-Dependent DNA Methylation
DNMT3A	DNA Methyltransferase 3A
GBM	Glioblastoma Multiforme
AK	Actinic Keratosis
NEAT1	Nuclear Paraspeckle Assembly Transcript 1
RT-PCR	Real-time Polymerase Chain Reaction
3′ UTRs	3′ Untranslated Regions
TRABD	TraB Domain Containing
