# Supplementary material for: Identifying an lncRNA-Related ceRNA Network to Reveal Novel Targets for a Cutaneous Squamous Cell Carcinoma
Source: Biology (Basel). 2021 May 13;10(5):432. doi: 10.3390/biology10050432 (PMC8152267; doi:10.3390/biology10050432)
Supplement: Supplementary file 1 [file biology-10-00432-s001.zip › Figure S1.pdf]

biology

MDPI

Article

Identifying an lncRNA-Related ceRNA Network to Reveal Novel Targets for a Cutaneous Squamous Cell Carcinoma

Yaqin Xu, Yingying Dong, Yunhua Deng, Qianrong Qi, Mi Wu, Hongmei Liang, Qiuyun She and Qing Guo

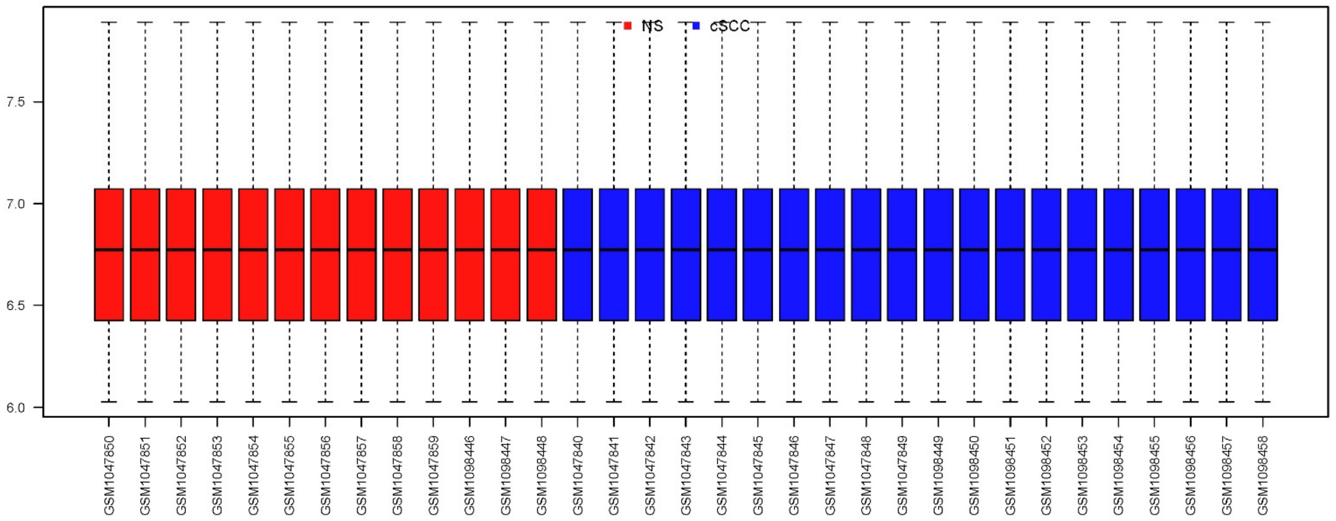

a

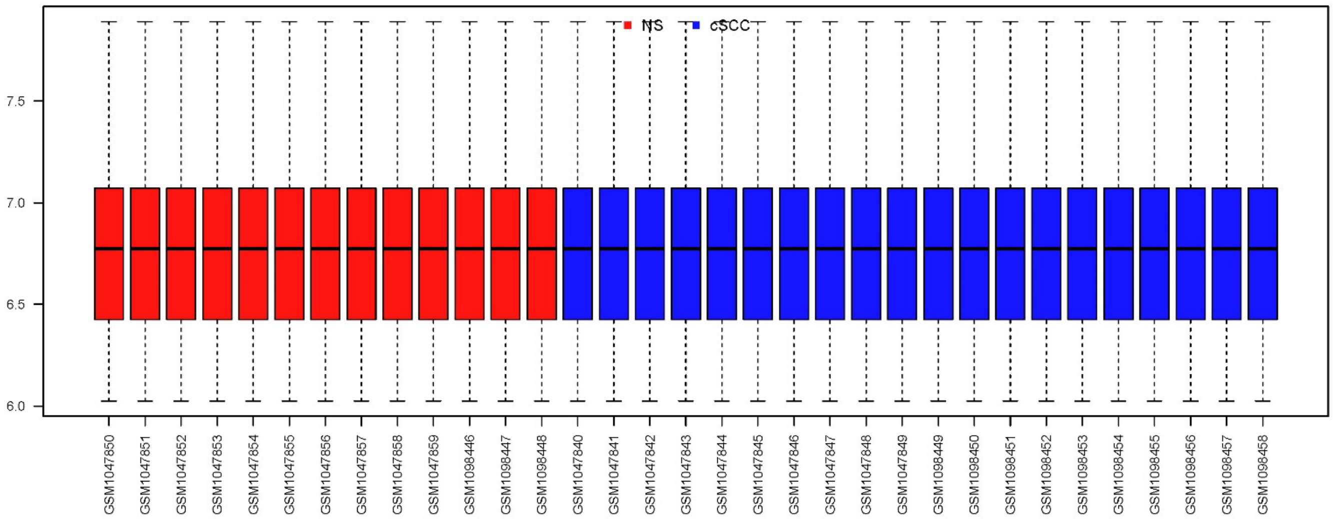

b

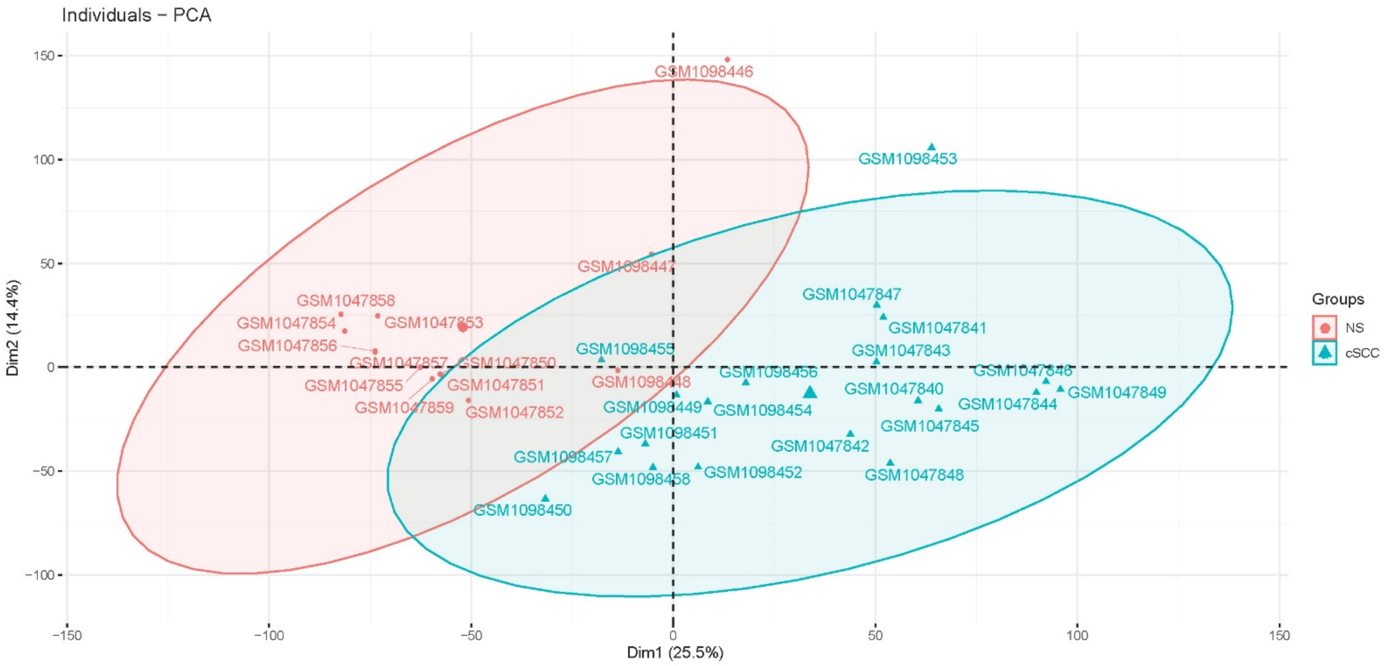

Individuals – PCA
Dim1 (25.5%)
Dim2 (14.4%)
Groups
NS
cSCC
GSM1098446
GSM1098453
GSM1098447
GSM1047858
GSM1047854
GSM1047853
GSM1047856
GSM1047857
GSM1047850
GSM1047855
GSM1047851
GSM1047859
GSM1047852
GSM1098448
GSM1098455
GSM1047847
GSM1047841
GSM1047843
GSM1047846
GSM1047849
GSM1098456
GSM1047840
GSM1047844
GSM1098449
GSM1098454
GSM1047845
GSM1098451
GSM1047842
GSM1098457
GSM1098458
GSM1098452
GSM1047848
GSM1098450

c

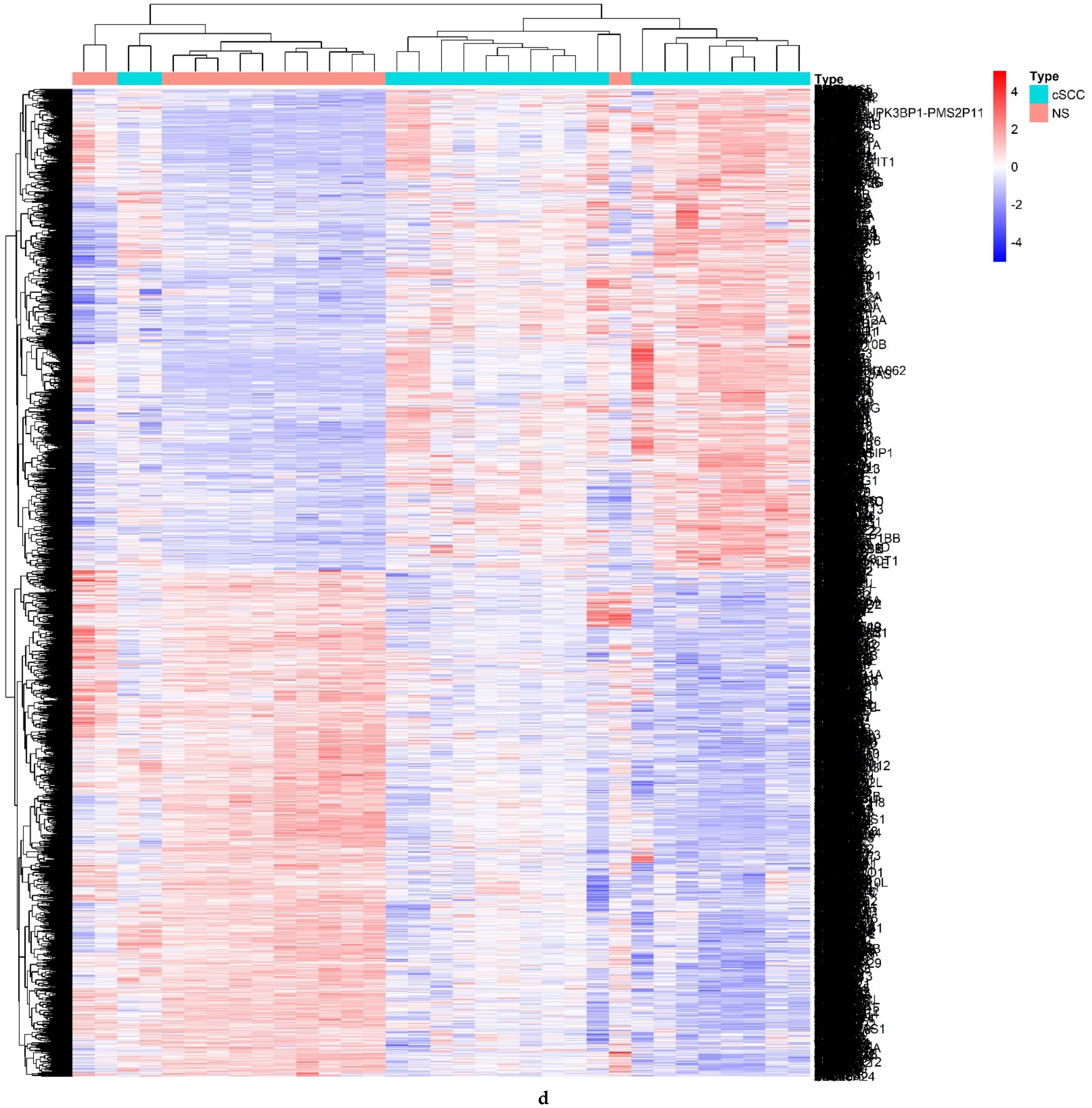

d

Figure S1. Data preprocessing. (**a**) Boxplot for combined datasets of GSE42677 and GSE45164 after quantile normalization without removing batch-effect; (**b**) Boxplot for combined datasets of GSE42677 and GSE45164 after quantile normalization with removing batch-effect; (**c**) The principal component analysis (PCA) of 20 cSCC and 13 NS tissues. (**d**) The cluster heat map of DEGs between 20 cSCC and 13 NS tissues.
